# The effect of maternal sleep quality in late pregnancy on prenatal, birth and early postnatal outcomes

**DOI:** 10.1111/jsr.14218

**Published:** 2024-04-16

**Authors:** Halime Abay, Begüm Öztürk Gülmez, Sena Kaplan

**Affiliations:** ^1^ Nursing Department, Faculty of Health Sciences Ankara Yıldırım Beyazıt University Çubuk Türkiye; ^2^ Nursing Department, Faculty of Medicine Research and Application Hospital Sivas Cumhuriyet University Sivas Türkiye

**Keywords:** birth experience, late pregnancy, maternal sleep quality, prenatal–birth–postnatal outcomes

## Abstract

This cross‐sectional study investigated the effect of maternal sleep quality in late pregnancy on prenatal, birth and early postnatal outcomes. The research was conducted in three parts with women at 28 or more weeks of gestation. In the first part, pregnant women admitted for delivery were evaluated in terms of eligibility criteria. Pregnant women in the latent phase of labour completed the Prenatal Questionnaire. In the second part, labour follow‐up was performed. The researchers completed the Birth Follow‐up Questionnaire based on observations and hospital birth records. In the third part, mothers were interviewed between the 12th and 24th hour of postnatal. Participants filled out the Postnatal Questionnaire, the Childbirth Experience Questionnaire, and the Pittsburgh Sleep Quality Index (*n* = 385). The data were analysed using the chi‐square independence test, Fisher test, independent samples *t*‐test, effect sizes, and binary logistic regression analysis. Participants had a mean maternal sleep quality score of 4.00 ± 1.38. They slept for 7.53 ± 0.92 hr on average. One‐third of the participants were poor sleepers (32.2%). Employed participants were 71.6% less likely to have poor maternal sleep quality than their non‐employed counterparts (odds ratio = 0.29, 95% confidence interval: 0.13–0.62; *p = 0*.002). The odds of poor maternal sleep quality increased by 13.7% when maternal weight gain during pregnancy increased by 1 kg (odds ratio = 1.14, 95% confidence interval: 1.03–1.26; *p = 0*.014). Increased maternal sleep quality positively affected the birth process (*p <* 0.05). Healthcare professionals should routinely screen the maternal sleep quality of pregnant women and increase their sleep hygiene.

## 1 INTRODUCTION

Sleep is a basic physiological need for physical and psychological health. Sleep quality is linked to variations in both the duration and quality of sleep, and the occurrence of symptoms experienced during sleep (Du et al., 2021). All sleep disorders increase the risk of poor sleep quality (American Academy of Sleep Medicine, 2023). Pregnancy is a natural process imbued with physiological and psychological changes. Nausea and vomiting, nocturia, reflux, foetal movements, fatigue, back and lower back pain, muscle cramps, restless leg syndrome, snoring, and labour stress associated with hormonal changes during pregnancy negatively affect sleep quality (Marinelli et al., 2022; Wang et al., 2022). Particularly in late pregnancy, when the maternal burden increases, there is a disruption in the sleep routine and a decrease in sleep duration. Moreover, it becomes more challenging to both fall asleep and maintain sleep (Beebe & Lee, 2007; Chang et al., 2010; Sedov et al., 2018). The prevalence of poor maternal sleep quality during pregnancy varies significantly, ranging from 16.9% to as high as 76.3% (Christian et al., 2019; Gelaye et al., 2017). A meta‐analysis reported this prevalence as 45.7% (Sedov et al., 2018).

Poor maternal sleep quality causes various disorders during the prenatal period (Antony et al., 2014; Cai et al., 2017; Ding et al., 2014; Li et al., 2017; Liu et al., 2021; Naghi et al., 2011; Wang et al., 2022). Poor maternal sleep quality also affects birth and postnatal outcomes (Du et al., 2021; Liu et al., 2021; Marinelli et al., 2022; Nakahara et al., 2020; Wang et al., 2022). Research shows that sleep‐disordered breathing, such as obstructive sleep apnea, can result in preterm delivery, low birth weight, low Apgar score, and the need for neonatal intensive care (Ding et al., 2014; Wang et al., 2022). Beebe & Lee (2007) reported that sleep quality was lowest the night before hospitalization for delivery. Poor sleep quality increases pain sensitivity and fatigue (Owusu et al., 2013) and prolongs labour (Naghi et al., 2011), adversely affecting birth experiences. However, no researchers have investigated the effect of maternal sleep quality on birth experiences.

Enhancing maternal sleep quality is crucial for promoting a healthy pregnancy and smooth delivery, contributing significantly to the well‐being of both the mother and the newborn. Despite the high prevalence of poor maternal sleep quality during pregnancy, this is not adequately integrated into antenatal care services (Anbesaw et al., 2021). This study examined the impact of maternal sleep quality in late pregnancy on prenatal, birth and early postnatal outcomes.

### 1.1 Research questions

Q_1_. Does maternal sleep quality in late pregnancy affect prenatal outcomes?

Q_2_. Does maternal sleep quality in late pregnancy affect birth outcomes?

Q_3_. Does maternal sleep quality in late pregnancy affect postnatal outcomes?

## 2 METHODS

### 2.1 Research design and sampling

This cross‐sectional study was conducted between 1 July 2022 and 1 November 2022 in Türkiye. The study population consisted of all pregnant women admitted to the Maternity and Children's Hospital of the Sivas Cumhuriyet University. The sample size was calculated using the formula for an unknown population: [*n* = *z* ^2^ × *p*(1 − *p*)/*d* ^2^ = 1.96^2^ × 0.435(1 – 0.435)/0.05^2^] (Charan & Biswas, 2013; Gunduz et al., 2016). The results showed that a sample of 378 would be large enough to detect significant differences (0.05 level of significance and 5% sampling error). Four‐hundred and fifty pregnant women were contacted with the prediction that there might be dropout in the study. The sample consisted of 385 pregnant women. Participants were recruited using simple random sampling. The study adhered to the Strengthening the Reporting of Observational Studies in Epidemiology (STROBE) checklist (Cuschieri, 2019).

### 2.2 Criteria for inclusion and exclusion

The inclusion criteria were: (1) being 19–35 years old; (2) being in the 28 or more weeks of gestational age; (3) being in the latent phase of labour (with cervical dilatation of 1–4 cm) (World Health Organization, 2008); and (4) having a singleton live foetus. The exclusion criteria were: (1) receiving epidural anaesthesia or analgesic during labour; (2) failing to complete the data collection tools; and (3) withdrawing.

### 2.3 Data collection

The data were collected face‐to‐face using five forms.

The Prenatal Questionnaire (PreNQ) was prepared by the researchers. It consisted of two parts. The first part comprised questions on individual characteristics, maternal age, education degree, employment status, family income, sleep quality during the preconception period, etc. (Cai et al., 2017; Gelaye et al., 2017; Wang et al., 2022). Sleep quality during the preconceptional period was assessed using the Visual Analogue Scale (VAS) (Price et al., 1983). The scale comprises 10 marks, extending from 0 to 10, positioned from the left end (mark one) to the right end (mark 10). Scores on this scale range from 0, indicating the poorest sleep quality, to 10, representing the best sleep quality. The second part consisted of questions on pregnancy‐related characteristics (planned pregnancy [yes/no], presence of maternal and foetal disease, maternal smoking during pregnancy [yes/no], maternal weight gain [kg], sleep duration [hr] and sleep position during pregnancy, etc.) (Du et al., 2021; Sedov et al., 2018; Wang et al., 2022; Warland et al., 2018). Maternal diseases were defined as physician‐diagnosed gestastional diabetus mellitus (GDM), preeclampsia and hypo/hyperthyroidism, while foetal diseases were defined as intrauterine growth restriction (IUGR) and foetal distress (Liu et al., 2021). The going‐to‐sleep position was classified as the left side, supine, right side and tummy (O'Brien et al., 2019).

The Birth Follow‐Up Questionnaire (BFQ) was prepared to facilitate labour monitoring by the researchers. The questionnaire consisted of two parts. The first part comprised items on labour characteristics (week of labour, severity of pain during labour, labour duration, episiotomy status [yes/no], preterm birth, etc.) (Ding et al., 2014; Li et al., 2017). The intensity of pain during labour was measured using VAS (from 0 [no pain] to 10 [extreme pain]) when the cervical opening was 5 and 10 cm (Price et al., 1983). VAS‐pain score was calculated by averaging the two measurements. The duration of the active, second and third phases of labour was recorded in hours. The active phase of labour was defined as the period during which cervical dilation progresses from 5 cm to complete dilation at 10 cm. The second phase of labour was defined as the period from complete cervical dilation until the actual birth of the baby. The third phase of labour was defined as the time from the birth of the baby until the delivery of the placenta and its associated membranes (World Health Organization, 2008). The sum of these periods was defined as labour duration. Preterm birth was characterized as occurring before the completion of 37 weeks of gestation, determined by calculating the duration between the mother's last menstrual period and the baby's delivery date (Du et al., 2021). The second part consisted of items on foetal and neonatal characteristics (foetal heart rate [FHR], Apgar score, neonatal birth weight, etc.) (Ding et al., 2014; Li et al., 2017). The FHR was determined by averaging the values measured when the cervical dilatation was 5 and 10 cm. For the Apgar score, the scores recorded at postnatal first and fifth minutes were averaged. Neonatal birth weight was measured in grams by midwives. Measures at birth were extracted from hospital delivery records.

The researchers prepared the Postnatal Questionnaire (PostNQ). The questionnaire consisted of items on the mother and newborn in the early postnatal period (level of fatigue, need for intensive care of the newborn in the first 24 hr [yes/no], etc.) (Christian et al., 2019; Du et al., 2021; Liu et al., 2021; Marinelli et al., 2022; Wang et al., 2022). Maternal fatigue was measured using VAS (from 0 [no fatigue] to 10 [extreme fatigue]) at the 12th–24th hour postnatal (Price et al., 1983).

The Childbirth Experience Questionnaire (CEQ) was developed by Dencker et al. (2010). The questionnaire consists of 22 items and four subscales: (1) birth process (Cronbach's alpha score of 0.82); (2) professional assistance/support (Cronbach's alpha score of 0.88); (3) perceived security/memories (Cronbach's alpha score of 0.78); and (4) agreement in decisions (Cronbach's alpha score of 0.62). The first 19 items are rated on a four‐point Likert‐type scale. The last three items are assessed using VAS. Four items (3, 5, 8 and 20) are reverse‐scored. Higher scores indicate better childbirth experiences (Dencker et al., 2010). The questionnaire was adapted to Turkish by Mamuk et al. (2019). The Turkish version has a Cronbach's alpha score of 0.76 (Mamuk et al., 2019), which was 0.76 in the present study.

The Pittsburgh Sleep Quality Index (PSQI) was developed by Buysse et al. (1989). The index consists of four open‐ended questions and 20 items rated on a three‐point Likert‐type scale. The index has seven components. The sum of the scores of these seven components is the total score ranging from 0 to 21. Lower scores indicate better sleep quality. Those with a PSQI score of < 5 are considered good sleepers, while those with a PSQI score of ≥ 5 are considered poor sleepers (sensitivity of 89.6% and specificity of 86.5%). The original scale has a Cronbach's alpha score of 0.83 (Buysse et al., 1989). The index was adapted to Turkish by Ağargün et al. (1996). The Turkish version has a Cronbach's alpha score of 0.80 (Ağargün et al., 1996), which was 0.81 in the present study.

### 2.4 Procedure

**FIGURE 1:**
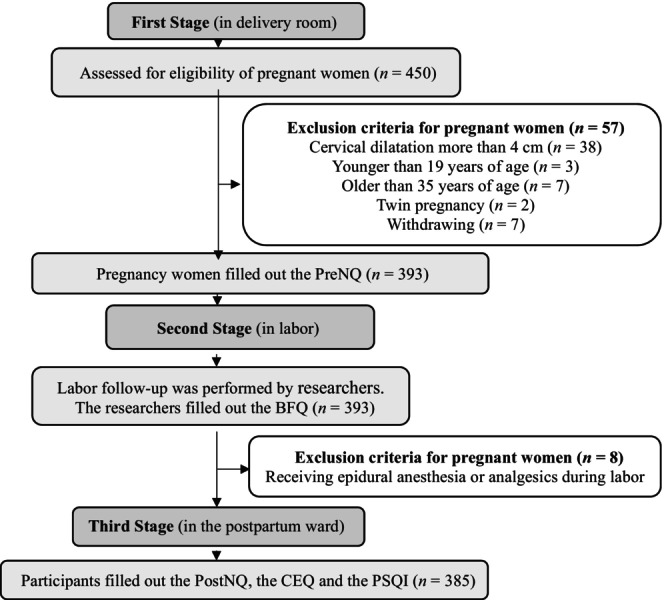
Flow chart. BFQ, Birth Follow‐Up Questionnaire; CEQ, Childbirth Experience Questionnaire; PostNQ, Postnatal Questionnaire; PreNQ, Prenatal Questionnaire; PSQI, Pittsburgh Sleep Quality Index.

The study had three parts (Figure 1).

In the first part, pregnant women were admitted to the delivery room. Each pregnant woman underwent Non‐Stress Test (NST) and vaginal examination. Pregnant women in the latent phase with uterine contractions and cervical dilatation of 1–4 cm were identified. Their compliance with other inclusion criteria was assessed. Informed consent was obtained from all participants. They completed the PreNQ. Four‐hundred and fifty pregnant women were contacted. Fifty‐seven women were excluded (cervical dilatation more than 4 cm [*n* = 38], younger than 19 years old [*n* = 3], older than 35 years old [*n* = 7], twin pregnancy [*n* = 2], and withdrawing [*n* = 7]). The sample of the first part consisted of 393 participants.

In the second part, labour follow‐up was performed by researchers. They filled out the BFQ based on observations and hospital delivery records. Eight women were excluded (receiving epidural anaesthesia or analgesics during labour [*n* = 8]). The sample of the second part consisted of 385 participants.

In the third part, all participants were interviewed in the postpartum ward between the 12th and 24th hour after delivery. All participants filled out the PostNQ, the CEQ and the PSQI during the interviews. The sample of the third part consisted of 385 participants.

### 2.5 Ethical considerations

The study was approved by the ethics committee of Ankara Yıldırım Beyazıt University (Approval No: 05.04.2022‐07). Permission was obtained from the hospital (Approval No: 01.07.2022‐182728). The study adhered to the ethical guidelines outlined in the World Medical Association's Declaration of Helsinki. Participation in the research was voluntary, and all participants provided written informed consent before taking part.

### 2.6 Statistical analysis

The data were analysed using the Statistical Package for Social Sciences (IBM, SPSS, Statistics 28) at a significance level of 0.05. Frequencies (numbers, percentages, etc.) were used for categorical variables, while descriptive statistics (means, standard deviations, etc.) were used for numerical variables. Parametric tests were used because good maternal sleep quality and poor maternal sleep quality groups had more than 30 participants (Koh & Ahad, 2020; Kwak & Kim, 2017). Parametric tests provide more reliable results than non‐parametric tests (Sheskin, 2011). Chi‐square test of independence, Fisher's test and independent samples *t*‐test were used to compare the characteristics of the two groups. Effect sizes were calculated. Binary logistic regression analysis was applied to identify factors affecting groups of maternal sleep quality variables.

## 3 RESULTS

### 3.1 Individual characteristics

Participants had a mean age of 30.30 ± 6.16 years. More than a quarter of the participants had primary school degrees (36.6%). More than a quarter of the participants had high school degrees (38.7%). Less than a quarter of the participants had bachelor's or higher degrees (24.7%). All participants lived in cities (100%). Most participants were not employed (84.9%). Less than half of the participants presenting for delivery at 36–40 weeks of gestation were at 39 weeks of gestation (45.2%). More than half of the participants had planned pregnancies (64.9%). Most participants had no chronic diseases during pregnancy (93.3%). Participants gained more than 9 kg on average (9.82 ± 2.10 kg). Most participants did not smoke during pregnancy (88.6%; not shown in table).

Most participants reported sleeping in the right lateral position (95.8%). Most participants had a sleep quality VAS score of ≥ 5 (min = 1, max = 10) during the preconceptional period (98.7%). More than half of the participants had a sleep quality VAS score of < 5 (min = 5, max = 8) during pregnancy (67.7%). It took participants 16.00 ± 7.03 min to fall asleep at night during the last month of their pregnancy. Participants slept for 7.53 ± 0.92 hr at night. More than half of the participants were good sleepers (67.8%), while more than a quarter of the participants were poor sleepers (32.2%; not shown in table). Participants had a mean PSQI score of 4.00 ± 1.38 (min = 1, max = 8; Table 1).

**TABLE 1:** PSQI and CEQ scores (*n* = 385)

Scales and subscales	Mean ± SD	Min–max	Scales' min–max
PSQI^a^	4.00 ± 1.38	1–8	0–21
Subjective sleep quality	0.94 ± 0.24	0–1	0–3
Sleep latency	0.64 ± 0.48	0–1	0–3
Sleep duration	0.13 ± 0.35	0–2	0–3
Habitual sleep efficiency	0.97 ± 1.01	0–3	0–3
Sleep disturbances	1.32 ± 0.47	1–2	0–3
Use of sleeping medication	0.00 ± 0.00	0–0	0–3
Daytime dysfunction	0.00 ± 0.05	0–1	0–3
CEQ^b^	42.36 ± 2.47	23–48	22–88
Birth process	14.19 ± 2.78	10–22	8–32
Professional assistance/support	6.00 ± 0.00	6–6	5–20
Perceived security/memories	16.61 ± 2.57	10–20	6–24
Agreement in decisions	5.70 ± 0.46	5–6	3–12

Abbreviations: CEQ, Childbirth Experience Questionnaire; PSQI, Pittsburgh Sleep Quality Index; SD, standard deviation.

^a^ Lower scores indicate better sleep quality. PSQI score < 5 good sleeper, ≥ 5 poor sleepers.

^b^ Higher scores indicate better childbirth experiences.

Table 2 shows the effect of some variables on maternal sleep quality. Most employed participants had good sleep quality (86.2%). More than half of the unemployed participants had good sleep quality (64.5%; *p =* 0.001). According to logistic regression, employed participants were 71.6% less likely to have poor maternal sleep quality than their unemployed counterparts (odds ratio [OR] = 0.29, 95% confidence interval [CI]: 0.13–0.62; *p = 0*.002; Table 4). This model had a correct classification rate of 67.3% and a Nagelkerke *R* ^2^ of 0.064. Most variables (maternal age, education degree, family income, sleep quality during preconceptional period, planned pregnancy, chronic disease during pregnancy, smoking during pregnancy, and maternal going‐to‐sleep position during pregnancy) did not affect participants' PSQI scores (*p >* 0.05). The effect sizes (Table 2) showed that other factors affected maternal sleep quality at a low level.

**TABLE 2:** PSQI scores by some variables (*n* = 385)

Variables	PSQI (sleep quality)^a^				Analysis	*p*	ES
	Good (*n* = 261)		Poor (*n* = 124)				
	*n*	%	*n*	%			
Age, years (Mean ± SD: 30.30 ± 6.16)							
< 30	129	67.89	61	32.11	0.002^b^	0.966	0.002
≥ 30	132	67.69	63	32.31			
Educational status, degree							
≤ High school	199	68.62	91	31.38	0.369^b^	0.543	0.031
≥ Bachelor's	62	65.26	33	34.74			
Employment status							
Yes	50	86.21	8	13.79	10.605^b^	0.001*	0.166
No	211	64.53	116	35.47			
Family income status							
Good	51	77.27	15	22.73	4.565^b^	0.102	0.109
Moderate	178	67.17	87	32.83			
Poor	32	59.26	22	40.74			
Sleep quality during preconceptional period, VAS score^c^ (Mean ± SD: 7.69 ± 1.64)							
< 5	5	100.00	–	–	2.400^d^	0.180	–
≥ 5	256	67.37	124	32.63			
Planned pregnancy status							
Yes	176	70.40	74	29.60	2.221^b^	0.136	0.076
No	85	62.96	50	37.04			
Chronic disease during pregnancy							
Yes	15	57.69	11	42.31	1.303^b^	0.280	0.058
No	246	68.52	113	31.48			
Smoking during pregnancy, cigarettes per day (Mean ± SD: 3.59 ± 1.92)							
Yes	28	63.64	16	36.36	0.393^b^	0.531	0.032
No	233	68.33	108	31.67			
Maternal going‐to‐sleep position during pregnancy							
Right side	249	67.48	120	32.52	0.397^d^	0.529	–
Supine	12	75.00	4	25.00			

Abbreviations: ES, effect size (Cohen‐w); PSQI, Pittsburgh Sleep Quality Index; SD, standard deviation; VAS, Visual Analogue Scale.

* *p <* 0.001.

^a^ Lower scores indicate better sleep quality. PSQI score < 5 good sleeper, ≥ 5 poor sleepers.

^b^ Chi‐square independence test.

^c^ VAS score (from 0 [worst sleep quality] to 10 [best sleep quality]).

^d^ Fisher test.

### 3.2 Prenatal, birth and postnatal outcomes

Table 3 compares the two groups' prenatal, birth and postnatal outcomes. Good sleepers gained 9.64 ± 2.04 kg during pregnancy, while poor sleepers gained 10.19 ± 2.19 kg during pregnancy (*p =* 0.016). According to the logistic regression analysis, the odds of maternal sleep quality being poor increased by 13.7% when maternal weight gain during pregnancy increased by one unit (OR = 1.14, 95% CI: 1.03–1.26; *p = 0*.014; Table 4). Good and poor sleepers had a mean CEQ birth process subscale score of 14.38 ± 2.93 and 13.77 ± 2.39, respectively (*p <* 0.05). There was no significant difference in other CEQ subscale scores between the groups (*p >* 0.05; Table 5). GDM, preeclampsia, hypo/hyperthyroidism, IUGR, foetal distress, episiotomy, preterm birth and neonatal intensive care needs had no effect on PSQI sleep quality scores (*p >* 0.05). Moreover, there was no significant difference in the week of labour, FHR in labour, pain in labour, duration of labour, neonatal Apgar score, neonatal weight, postnatal fatigue between good and poor sleepers (*p >* 0.05; Table 3). The effect sizes showed that the other factors affected maternal sleep quality at a low level (Tables 3 and 5).

**TABLE 3:** PSQI scores by prenatal, birth and postnatal outcomes (*n* = 385)

Outcomes	PSQI (sleep quality)^a^				Analysis	*p*	ES
	Good (*n* = 261)		Poor (*n* = 124)				
	*n*	%	*n*	%			
GDM							
Yes	8	72.73	3	27.27	0.126^b^	0.504	0.018
No	253	67.65	121	32.35			
Preeclampsia							
Yes	5	50.00	5	50.00	1.488^b^	0.303	0.062
No	256	68.27	119	31.73			
Hypo/hyperthyroidism							
Yes	2	40.00	3	60.00	1.792^c^	0.334	0.068
No	259	68.16	121	31.84			
IUGR							
Yes	1	50.00	1	50.00	0.291^b^	0.541	0.027
No	260	67.89	123	32.11			
Foetal distress							
Yes	11	78.57	3	21.43	0.773^b^	0.562	0.045
No	250	67.39	121	32.61			
Episiotomy							
Yes	132	66.00	68	34.00	0.612^c^	0.434	0.040
No	129	69.73	56	30.27			
Preterm birth (< 37 gestational weeks)							
Yes	24	68.6	11	31.4	0^b^	0.918	0.048
No	237	67.7	113	32.3			
Neonatal intensive care needs in the first 24 hr							
Yes	12	75.00	4	25.00	0.397^c^	0.529	0.032
No	249	67.48	120	32.52			
	Mean	SD	Mean	SD			
Maternal weight gain during pregnancy, kg	9.64	2.04	10.19	2.19	−2.413^d^	0.016*	0.260
Birth time, week	38.31	1.08	38.30	1.04	0.136^d^	0.446	0.009
FHR in labor^e^, /dk	135.64	2.04	135.42	2.10	0.989^d^	0.323	0.107
VAS‐pain in labour^f^	7.30	0.19	7.32	0.18	−0.888^d^	0.375	−0.096
Duration of labour, hr	9.94	1.92	9.70	1.92	1.150^d^	0.125	0.126
Apgar score^g^	8.08	0.78	8.06	0.80	0.210^d^	0.834	0.024
Neonatal birth weight, g	3041.68	428.30	3088.87	463.97	−0.983^d^	0.326	−0.106
VAS‐postnatal fatigue^h^	8.79	0.96	8.85	1.00	−0.580^d^	0.562	−0.063

Abbreviations: ES, effect size (Cohen‐w); FHR, foetal heart rate; GDM, gestational diabetes mellitus; IUGR, intrauterine growth restriction; PSQI, Pittsburgh Sleep Quality Index; SD, standard deviation; VAS, Visual Analogue Scale.

* *p <* 0.05.

^a^ Lower scores indicate better sleep quality. PSQI score < 5 good sleeper, ≥ 5 poor sleepers.

^b^ Fisher test.

^c^ Chi‐square independence test.

^d^ Independent samples *t*‐test.

^e^ Averaging the values measured when the cervical dilatation was 5 and 10 cm.

^f^ VAS score for pain in labour (from 0 [no pain] to 10 [worst pain]). Averaging the values measured when the cervical dilatation was 5 and 10 cm.

^g^ Averaging the values recorded at postnatal first and fifth minutes.

^h^ VAS score for fatigue in postnatal 12th–24th hour (from 0 [no fatigue] to 10 [extreme fatigue]).

**TABLE 4:** Multiple regression model by maternal sleep quality variable.

Variables	Β	SE	Wald	d.f.	*p*	OR	95% CI	
							Lower	Upper
Maternal employment status (Yes)	−1.260	0.401	9.886	1.000	0.002*	0.284	0.129	0.622
Ref (No)								
Maternal weight gain during pregnancy	0.128	0.052	6.038	1.000	0.014*	1.137	1.026	1.259
CEQ‐Birth process subscale	−0.008	0.045	0.028	1.000	0.866	0.993	0.909	1.083
Constant term	−1.545	1.982	0.608	1.000	0.436	0.213		

Abbreviations: CEQ, Childbirth Experience Questionnaire; CI, confidence interval; d.f., degrees of freedom; OR, odds ratio.

* *p <* 0.05.

**TABLE 5:** PSQI scores by CEQ scores (*n* = 385)

Scale and subscales	PSQI (sleep quality)^a^				Analysis	*p*	ES
	Good (*n* = 261)		Poor (*n* = 124)				
	Mean	SD	Mean	SD			
CEQ^b^	42.40	2.67	42.27	1.99	0.461^c^	0.645	0.053
Birth process	14.38	2.93	13.77	2.38	2.187^c^	0.030*	0.230
Professional assistance/support	6.00	0.00	6.00	0.00	–	–	–
Perceived security/memories	16.52	2.52	16.80	2.66	−0.995^c^	0.320	−0.108
Agreement in decisions	5.69	0.46	5.71	0.46	−0.398^c^	0.691	−0.043

Abbreviations: CEQ, Childbirth Experience Questionnaire; ES, effect size (Cohen‐w); PSQI, Pittsburgh Sleep Quality Index; SD, standard deviation.

* *p <* 0.05.

^a^ Lower scores indicate better sleep quality. PSQI score < 5 good sleeper, ≥ 5 poor sleepers.

^b^ Higher scores indicate better childbirth experiences.

^c^ Independent samples *t*‐test.

## 4 DISCUSSION

This study had four key findings. First, one in three pregnant women in late pregnancy were poor sleepers. Second, maternal employment affected sleep quality, and maternal sleep quality affected only gestational weight gain among prenatal outcomes. Third, the increase in maternal sleep quality positively affected only the birth process subscale score among birth outcomes. Fourth, maternal sleep quality had no effect on postnatal outcomes.

A new systematic review showed that sleep problems increase, especially in the third trimester of pregnancy. During this period, the prevalence of insomnia was 42.4%. Moreover, one in three pregnant women failed to maintain enough sleep (Salari et al., 2021). Therefore, we evaluated maternal sleep quality in the last month before delivery. The average PSQI score was 4.00. The prevalence of poor sleepers (PSQI score ≥ 5) was 32.2%. The prevalence of poor maternal sleep quality is 17.0% in Peru (Gelaye et al., 2017), 30.8% in Ethiopia (Anbesaw et al., 2021), 41.2% in Vietnam (Huong et al., 2019), 59.0% in the USA (Christian et al., 2019) and 77.0% in Iran (Ahmadi et al., 2019). This wide range suggested that socioeconomic, socio‐cultural and demographic characteristics might be at play (Anbesaw et al., 2021). However, the significant difference in the prevalence of poor sleepers in studies conducted in the same country refuted this argument. For example, in a study conducted in China, the prevalence of poor sleepers was 15.3% (Xu et al., 2017), which was 34.1% (Du et al., 2021) and 54.2% (Yang et al., 2020) in the other two studies. Sedov et al. (2018) reported that the mean PSQI score increased by 1.68 points from the second to the third trimester (Sedov et al., 2018). This result suggested that the trimester of pregnancy in which maternal sleep quality is assessed might affect the prevalence of poor sleepers. Researchers have focused on early pregnancy (Du et al., 2021), 24th and 28th gestational weeks (Gelaye et al., 2017), all trimesters (Anbesaw et al., 2021), or only the third trimester (Ahmadi et al., 2019). The difference in the prevalence of poor maternal sleep quality could be attributed to the gestational period. However, Tsai et al. (2016) divided the prevalence of poor maternal sleep quality into early, middle and late pregnancy periods but did not reach this conclusion (43.3%, 37.2% and 50.6%, respectively; Tsai et al., 2016). Mindell et al. (2015) also examined the prevalence of poor maternal sleep quality by months in pregnancy and found inconsistencies in the rates (74.0%, 76.2%, 72.0%, 72.5%, 72.5%, 72.8%, 75.1% and 83.5% at less than 2 months, 3rd, 4th, 4th, 5th, 6th, 7th and above 8 months, respectively; Mindell et al., 2015). Therefore, the reasons for these differences in prevalence may be the research design, inclusion criteria, sampling technique and size, and differences in diagnostic criteria.

Research also suggests that age, education, family income and tobacco use are not significantly associated with sleep quality (Conlon et al., 2021; Wang et al., 2022). On the other hand, maternal employment status affects sleep quality and is associated with unemployment excessive sleep duration (Signal et al., 2014; Tsai et al., 2016; Xu et al., 2017). Long sleep duration disrupts circadian rhythm and reduces maternal sleep quality (Wang et al., 2022). Our results also showed that only maternal employment status affected sleep quality. High levels of progesterone during pregnancy make women feel extremely sleepy during the day (Wang et al., 2022). We believe that employed pregnant women maintain a consistent wake‐up time in the morning and avoid daytime napping, which helps preserve their biological rhythm, ultimately positively impacting their sleep quality.

Changes in neuroendocrine function and metabolism caused by long‐term sleep disorders result in insulin resistance and obesity (Mesarwi et al., 2013). Especially breathing‐related sleep disorder is a risk factor for high gestational weight gain (Izci‐Balserak & Pien, 2010). Our results showed that poor sleepers gained more weight than good sleepers during pregnancy, and that 1 kg of weight gain during pregnancy increased the odds of poor maternal sleep quality by 13.7%. Therefore, sleep hygiene and lifestyle changes such as going to sleep and waking up at the same time, avoiding alcohol, caffeine and nicotine, taking a warm shower before going to sleep, and exercising daily improve maternal sleep quality and facilitate the control of gestational weight gain (Nodine & Matthews, 2013).

The prenatal characteristics of our cohort highlight outcomes that indicate a healthy pregnancy. In addition, most of our participants sleep on the right side during pregnancy. Adopting a left‐side sleeping position during pregnancy alleviates pressure on uterine blood vessels, enhances blood flow to the uterus and subsequently lowers the risk of adverse prenatal outcomes. However, recent research has shown no significant impact on prenatal and birth outcomes (preeclampsia, IUGR or stillbirth) when sleeping on the back or right side compared with sleeping on the left side during pregnancy (Dunietz et al., 2020; Silver et al., 2019). Our participants slept about 7 hr at night. Research shows that pregnant women who sleep 6.0–7.9 hr at night have better birth outcomes (low birth weight infants, small for gestational age infants, etc.) than those who sleep 9.0–9.9 hr at night (Marinelli et al., 2022; Murata et al., 2021). Our participants were admitted to the delivery room, earliest at 36 weeks of gestation. Approximately half of our participants were admitted to the delivery room at 39 weeks of gestation. Our sample comprised women who: (1) did not experience sleep issues before pregnancy; (2) enjoyed a healthy pregnancy marked by high maternal sleep quality; and (3) had no risk of preterm delivery. Our participants did not have any indication for Caesarean section after admission to the delivery room, which indicates that they had healthy pregnancy and delivery processes. Our participants were less likely to experience adverse prenatal, birth and postnatal outcomes because they had very low‐risk pregnancies. While some researchers state that poor maternal sleep quality has no effect on preeclampsia, GDM, preterm birth and low birth weight (Li et al., 2017; Nakahara et al., 2020; Wang et al., 2022), others state that it is associated with adverse prenatal, birth and postnatal outcomes (Cai et al., 2017; Ding et al., 2014; Li et al., 2017; Liu et al., 2021; Naghi et al., 2011; Wang et al., 2022). In fact, Warland et al. (2018) reported that obstructive sleep apnea has a negative impact on foetal viability (Warland et al., 2018). It is possible that the degree of sleep problems present in our sample was not severe enough to affect foetal circulation. This uncertainty can be reduced by conducting evidence‐based, multicentre and prospective cohort studies with controlled confounders and large samples.

The night before hospitalization for delivery marks the lowest maternal sleep quality. This decline in sleep quality corresponds to a reduced pain threshold, rendering pregnant women more susceptible to experiencing heightened labour pain (Beebe & Lee, 2007). Research shows that poor sleepers tend to endure a prolonged labour process (Naghi et al., 2011), exhibit increased fatigue throughout labour, and report higher levels of fear and anxiety (Chang et al., 2010) compared with those classified as good sleepers. Our results showed a significant difference between good and poor sleepers' CEQ “birth process” subscale scores. The birth process subscale assesses women's sense of personal control, feelings about the birth process, and labour pain (Dencker et al., 2010; Mamuk et al., 2019). Our results showed that poor sleepers had a significantly lower mean CEQ “birth process” subscale score than good sleepers. Our results also showed a positive correlation between maternal sleep quality and CEQ “birth process” subscale scores. However, there was no significant difference in perceived labour pain, episiotomy application, and duration of labour between good and poor sleepers. It is noteworthy that participants with low‐risk pregnancies had negative birth experiences, which may have something to do with vaginal delivery procedures and national health policies.

### 4.1 Limitations

The study has four limitations. First, the researchers relied on self‐reports rather than using objective medical methods to assess maternal sleep quality. Second, the researchers knew nothing about participants' sleep disorders because they were left undiagnosed. Third, the researchers could not investigate the impact of maternal sleep quality on the mode of delivery because none of the participants had indications for C‐section. Fourth, the results are sample‐specific and cannot be generalized to all pregnant women and mothers.

## 5 CONCLUSION

While approximately one in three pregnant women experience poor sleep quality, it appears that this factor does not significantly impact the majority of prenatal, birth and early postnatal outcomes. Women's participation in work life positively affects maternal sleep quality. Good maternal sleep quality positively supports the birth process. Therefore, the following suggestions can be made. First, researchers should conduct further evidence‐based research on this topic. Second, researchers should use objective medical methods to assess maternal sleep quality. Third, women should participate more in the workforce. Fourth, as part of antenatal care, healthcare professionals should routinely screen women for maternal sleep quality and sleep patterns from early pregnancy. Fifth, health professionals should view pregnancy as an opportunity to teach women about sleep hygiene habits.

## AUTHOR CONTRIBUTIONS

**Halime Abay:** Conceptualization; investigation; writing – original draft; methodology; writing – review and editing; software; formal analysis; validation; visualization; project administration; resources; funding acquisition. **Begüm Öztürk Gülmez:** Investigation; data curation; software; formal analysis; resources; funding acquisition; methodology; conceptualization. **Sena Kaplan:** Supervision; methodology; conceptualization; writing – review and editing; software; formal analysis; validation; resources; funding acquisition; investigation.

## FUNDING INFORMATION

This research did not receive any specific grant from funding agencies in the public, commercial or not‐for‐profit sectors.

## CONFLICT OF INTEREST STATEMENT

The authors declare that they have no known competing financial interests or personal relationships that could have appeared to influence the work reported in this paper. The authors declare that they do not have any conflicts of interest.

## PATIENT CONSENT STATEMENT

Participation was voluntary. Informed consent was obtained from all participants included in this study.

## Data Availability

The data that support the findings of this study are available from the corresponding author upon reasonable request.
